# Triglyceride-glucose index as a novel predictor of major adverse cardiovascular events in patients with coronary revascularization: a meta-analysis of cohort studies

**DOI:** 10.1080/07853890.2025.2607796

**Published:** 2025-12-27

**Authors:** Chunyu Zhang, Minghao Li, Lin Liu, Yi Zhong, Yulei Xie, Bin Liao, Jian Feng, Li Deng

**Affiliations:** ^a^ Department of Cardiology, The Affiliated Hospital of Southwest Medical University, Stem Cell Immunity and Regeneration Key Laboratory of Luzhou, Luzhou, China; ^b^ School of Rehabilitation, Capital Medical University, Beijing, China; ^c^ Department of Rehabilitation Medicine, Affiliated Hospital of North Sichuan Medical College, Sichuan, China; ^d^ Department of Cardiovascular Surgery, The Affiliated Hospital of Southwest Medical University, Metabolic Vascular Diseases Key Laboratory of Sichuan Province, Luzhou, China; ^e^ Department of Rheumatology, The Affiliated Hospital of Southwest Medical University, Luzhou, China

**Keywords:** Triglyceride-glucose index, coronary revascularization, meta-analysis, cardiovascular disease risk

## Abstract

**Background:**

The triglyceride-glucose index (TyG) has gained attention as an alternative indicator for assessing insulin resistance (IR). The purpose of this study was to comprehensively summarize the correlation between the TyG index and cardiovascular events in patients with coronary revascularization.

**Methods:**

PubMed, Web of Science, Embase, and The Cochrane Library databases were searched to find relevant literature on the prognostic assessment of TyG index in patients undergoing coronary artery revascularization. Utilize the risk ratio (RR) and its 95% confidence interval (CI) as the standard for assessing the correlation between TyG and major adverse cardiovascular events (MACEs) in patients undergoing coronary artery revascularization. Conduct sensitivity analysis and subgroup analysis to detect the sources of heterogeneity and assess the stability of the results.

**Results:**

A total of 12 studies involving 9,973 participants were included. The results of the study indicate that a high TyG index was related to the major adverse cardiovascular event in patients undergoing coronary artery revascularization (RR:2.0,95%CI: 1.71–2.35, *I^2^*=76.2%, *p* < 0.0001). Subgroup analysis reveals that the probability of MACEs occurring in patients with high TyG index is higher than in those with low TyG index after two different coronary artery revascularization procedures: CABG group (RR:2.10, 95%CI:1.80–2.45, I2 = 20.9%, *p* = 0.0001). PCI group: (RR:1.94, 95%CI:1.54–2.46, I2 = 84.2%, *p* < 0.00001). Additionally, we also demonstrated the prognostic value of the TyG index in all-cause mortality(*p* = 0.003), non-fatal myocardial infarction(*p* = 0.003), non-fatal stroke(*p* < 0.0001) and repeat revascularization(*p* < 0.0001).

**Conclusions:**

Higher TyG index may be independently associated with higher incidence of MACEs in patients with coronary revascularization.

## 1. Introduction

Coronary Artery Disease (CAD) stands as a prominent global contributor to disease and death [1], presenting substantial challenges and economic strains on public health and individuals [2]. Among these, Acute Coronary Syndrome (ACS) emerges as the most severe subtype of the condition. Coronary artery revascularization, encompassing percutaneous transluminal coronary intervention (PCI) and coronary artery bypass grafting (CABG), proves to be an effective approach in mitigating symptoms of ischemic heart disease and enhancing survival rates [3–5]. Nonetheless, owing to the intricate nature of coronary artery disease, patients undergoing coronary revascularization still face a higher risk of adverse cardiovascular events. Hence, the timely identification of post-coronary artery revascularization patients harbouring a lingering high risk proves pivotal for subsequent clinical interventions and reducing the occurrence of future cardiovascular events.

Simental introduced the triglyceride-glucose index in 2008 [6], defined by the formula TyG index = ln [Fasting triglyceride (mg/dL) × Fasting glucose (mg/dL)]/2 [6]. In clinical practice, it primarily serves as an alternative indicator for detecting insulin resistance (IR) [7]. In recent years, a large number of studies have demonstrated that an elevated TyG index is linked to a higher incidence of adverse cardiovascular events in patients with coronary heart disease [8–12]. Conversely, some studies have indicated that the TyG index does not correlate with the subsequent risk of cardiovascular diseases [13–15]. Presently, the controversy persists regarding the predictive role of the triglyceride-glucose index in cardiovascular diseases, coupled with the absence of dedicated studies on the applicability of TyG index in predicting major adverse cardiovascular events (MACEs) following coronary artery revascularization. Hence, this study seeks to perform a meta-analysis of previously published cohort studies, summarizing the potential correlation between TyG index and the subsequent incidence of MACEs in patients following coronary artery revascularization. Additionally, The predictive effect of TyG index on adverse cardiovascular events after different coronary revascularization was investigated by subgroup analysis. The goal is to furnish personalized treatment plans for early intervention in patients after coronary artery revascularization, thereby diminishing the incidence of adverse cardiovascular events.

## 2. Methods

We conducted this systemic review and meta-­analyses following the Preferred Reporting Items for Systematic Reviews and Meta-Analyses (PRISMA) guideline [16]. Our protocol was registered in PROSPERO (registration number: CRD42024498404).

### 2.1. Search strategy

The meta-analysis includes cohort studies examining the prediction of MACEs following coronary artery revascularization, utilizing the TyG index. We retrieved relevant literature investigating the prognostic values of the TyG index for MACEs and other adverse outcomes among patients undergoing coronary artery revascularization from four English databases: PubMed, Web of Science, Embase, and Cochrane Library. The search was conducted with a deadline set on November 20, 2024. We employed keywords such as ‘T/Gly index,’ ‘triglyceride-glucose index,’ ‘triglyceride and glucose index,’ ‘triglyceride glucose index,’ ‘triacylglycerol glucose index,’ ‘acute coronary syndrome,’ ‘percutaneous Coronary Intervention,’ ‘PCI,’ ‘Coronary Artery Bypass,’ ‘CABG,’ and ‘major adverse cardiovascular and cerebrovascular events’ in the search. Furthermore, we performed manual searches, including screening the reference lists of prior systematic reviews and meta-analyses, to identify pertinent articles for further analysis.

### 2.2. Inclusion and exclusion criteria

Literature screening was based on the following inclusion and exclusion criteria. The inclusion criteria: (1) Study type: Retrospective or prospective cohort studies; (2) Study population: Patients following coronary artery revascularization (PCI or CABG); (3) Participants exposed to varying levels of the TyG index at baseline; (4) Primary outcome measure: MACEs serve as the primary outcome, comprising all-cause death, non-fatal myocardial infarction, non-fatal stroke, repeat revascularization and cardiac rehospitalization. (5) Secondary outcome measures: All-cause death, non-fatal myocardial infarction, non-fatal stroke, and repeat revascularization.

The exclusion criteria: (1) Follow-up time less than 6 months; (2) Exclusion of cross-sectional studies, reviews, preclinical studies, and studies unrelated to the meta-analysis’s purpose; (3) Exclusion of animal experiments, conference papers, case reports, non-Chinese and non-English literature, and duplicate publications; (4) Studies that did not provide outcome indicators for MACEs after TyG grouping were excluded. (5) Exclude studies with sample size less than 300.

### 2.3. Data extraction and quality assessment

Two researchers (ZCY and LMH) independently performed data extraction and quality assessment for the included studies. Any disagreements were resolved through consensus, discussion, or negotiation involving a third researcher (LL).

The extracted data encompass: (1) First author’s name, publication year, and country; (2) Characteristics of study design; (3) Patient attributes, including diagnosis, sample size, age, gender, hypertension, diabetes, hyperlipidemia; (4) TyG index analysis model; (5) Follow-up time; (6) Outcomes, comprising MACEs, all-cause death, non-fatal myocardial infarction, non-fatal stroke, and repeat revascularization. In instances of insufficient information, we initiated contact with the respective authors. Disagreements were resolved through consultation with an expert in the field (J Feng), and judgments were made accordingly. The Newcastle-Ottawa Scale (NOS) was employed for assessing the quality of included studies and evaluating cohort studies based on three aspects: selection of study participants (4 stars); comparability of outcomes (2 stars); and quality of outcomes (3 stars). Studies meeting 7 or more stars were considered high-quality.

### 2.4. Statistical analysis

Utilizing STATA (Version 15.1) for statistical analysis. Employing the risk ratio (RR) and its corresponding 95% confidence intervals (CIs) as the metrics to assess the association between TyG index and MACE risk post-coronary artery revascularization Among the included literature, Qi Zhao a 2020, ZhenGuo Wu a 2023, and Shutong Dong 2023 used optimal cutoff points for grouping, while the remaining studies utilized tertiles or quartiles of TyG index in the study population. Consequently, the TyG index was treated as a categorical variable in this study and extracted the RR of MACEs of the group with the highest TyG index and the group with the lowest TyG index for analysis. Employing Cochrane’s Q test to evaluate heterogeneity among the included cohort studies and calculating the *I^2^* statistic [17]. If *I^2^* >50%, significant heterogeneity was deemed present. A random-effects model was employed to synthesize RR data, representing a more generalized approach capable of accommodating potential heterogeneity among included studies [18]. Performing sensitivity analysis by systematically excluding one study at a time to assess the robustness of the results [19]. Utilizing pre-specified subgroup analysis to evaluate the predictive role of the TyG index on MACEs in patients undergoing various revascularization procedures. Statistical significance was defined as a P value < 0.05. Evaluating potential publication bias through visually inspecting funnel plot symmetry and conducting Egger’s test [20].

## 3. Results

### 3.1. Study selection and study characteristics

**Figure 1.**
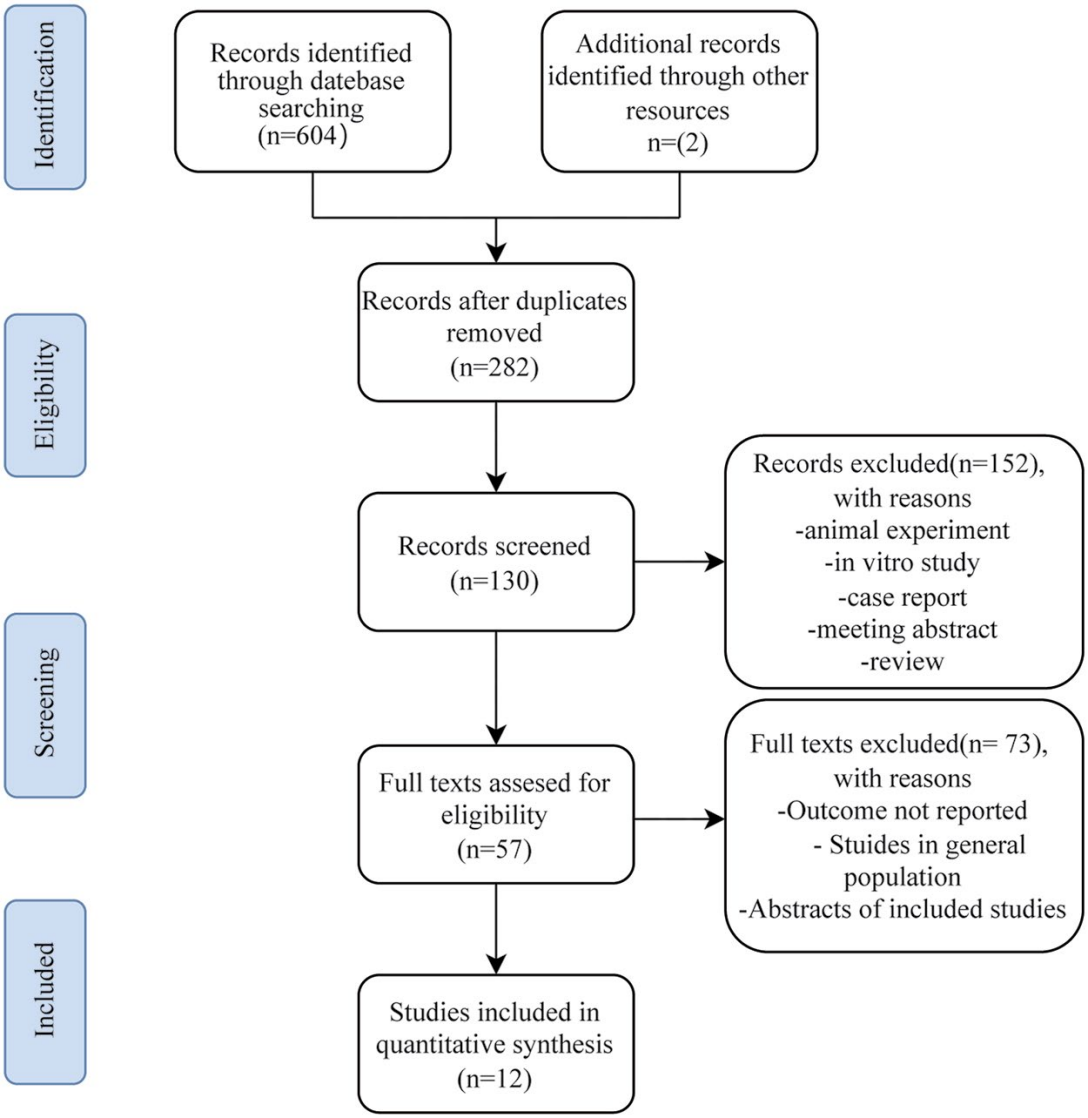
Flow diagram of the study selection process.

The pre-defined search strategy yielded 604 records from PubMed, Embase, Web of Science, and Cochrane databases. Two additional articles were identified through manual searches, and based on pre-defined inclusion and exclusion criteria, a total of 12 studies met the criteria for analysis [21–32]. Figure 1 depicts the study selection process and provides reasons for exclusion after full-text reading. Initially, 324 duplicate publications were removed using reference management software (EndNote X7). Subsequently, 152 articles were excluded due to their nature as animal experiments, case reports, reviews, or irrelevant content. Subsequently, 57 publications underwent full-text review. Following additional screening, 12 cohort studies, comprising 9973 participants, were included for subsequent meta-analysis. Within the 9973 participants, the average/median age ranged from 59.7 to 66.6 years. All included studies were conducted in China. TyG index cutoff values were determined through ROC analysis, the Youden index, tertiles, and quartiles. Table 1 presents the detailed characteristics of the included studies. Seven studies scored NOS ratings ranging from 7 to 8, signifying a low risk of bias according to the NOS scale. Five studies scored NOS ratings of 6, with the primary bias risk attributed to the lack of comparability due to the absence of adjusted confounding factors (Table 2).

**Table 1.** Characteristics of the included studies.

Study	Country	Year	Population	design	TyG Index analysis	TyG cut-off value	Age(mean + SD)	Sample size	Male	HTN%	DM%	HPL%	Follow-up Duration (months)	Outcome
Liang Chen	China	2022	CABG	RC	T3:TI	NA	62.9 ± 8.1	1052	891	72.15	NA	34.13	24months	①②③ ④⑤
He Zhang	China	2023	CABG	RC	High TyG: low TyG	9.17	66 ± 8.93	386	276	74.6	NA	NA	60months	①②③ ④⑤
Zhenguo Wu a	China	2023	CABG	RC	High TyG: low TyG	8.87	62.79 ± 8.18	1225	857	62.6	32.2	32.2	69months	①
Zhenguo Wu b	China	2023	CABG	RC	T3:TI	NA	62.65 ± 8.25	674	474	65	34.1	33.8	69months	①②③ ④⑤
Zhenguo Wu c	China	2023	CABG	RC	T3:TI	NA	62.73 ± 8.32	554	411	60.6	25.8	29.8	69months	①②③ ④⑤
Xiaoteng Ma	China	2020	PCI	RC	T3:TI	NA	61 ± 10.24	516	359	69.2	NA	79.8	11months	①②③ ④⑤
Qi Zhao a	China	2020	PCI	RC	High TyG: low TyG	9.18	60.9 ± 8.3	798	545	71.8	NA	89	11months	①② ④⑤
Qi Zhao b	China	2020	PCI	RC	High TyG: low TyG	8.72	59.7 ± 9.3	1510	1113	57.2	NA	84.5	48months	①②③ ④⑤
LUO	China	2019	PCI	RC	Q3:Q1	NA	63.2 ± 12.57	546	432	62.08	26.37	NA	12months	①
Shiqiang Xiong	China	2022	PCI	RC	T3: T1	NA	66.6 ± 11.66	659	463	64.03	36.1		30.72months	①②③ ④⑤
Shiyi Tao	China	2023	PCI	RC	T3:T1	NA	64.86 ± 11.63	895	654	74.3	45.3	67.9	20.4months	①
Shutong Dong	China	2023	PCI	RC	High TyG: low TyG	NA	64.5 ± 8.0	1158	872	72.3	46	99.5	42months	①②③ ④⑤

① MACEs, ② all-cause mortality, ③ non-fatal stroke, ④ non-fatal MI, ⑤ repeat revascularization.

RC: Retrospective cohort; Q: Quartile; T: Tertile; PCI: Percutaneous coronary intervention; TyG index: Triglyceride-glucose index; MACEs: Major adverse cardiovascular events; CABG: Coronary artery bypass graft; DM: Diabetes; HTN: Hypertension; HPL:Hyperlipidemia; RR:relative risk; CI: confidence interval.

**Table 2.** Details of study quality evaluation *via* the Newcastle-Ottawa scale.

	Selection					Outcome			
StudiesAuthor/Year	Representa­tiveness of the exposed cohort	Selection of the non-exposed cohort	Ascertainment of exposure	Outcome of interest was not present at start of study	Comparability	Assessment of outcome	Long enough follow-up for outcomes to occur	Adequacy of follow up of cohorts	Total
Liang Chen 2020	1	1	1	0	1	0	0	1	6
He Zhang 2022	1	1	1	1	0	0	1	1	7
Zhenguo Wu a 2023	1	1	0	1	1	1	1	1	8
Zhenguo Wu b 2023	1	1	0	1	1	1	1	1	8
Zhenguo Wu C 2023	1	0	1	0	1	0	1	1	6
Xiaoteng Ma 2020	1	1	0	1	1	1	1	1	8
Qi Zhao a 2020	1	0	1	0	1	1	1	1	7
Qi Zhao b 2020	1	1	1	1	1	0	1	1	8
Luo 2019	1	1	1	0	1	0	1	1	6
Shiqiang Xiong 2022	1	1	1	0	1	0	1	1	6
Shiyi Tao 2023	1	1	0	1	1	1	1	1	7
Shutong Dong 2023	1	0	0	1	1	1	1	1	6

### 3.2. Primary outcome

**Figure 2.**
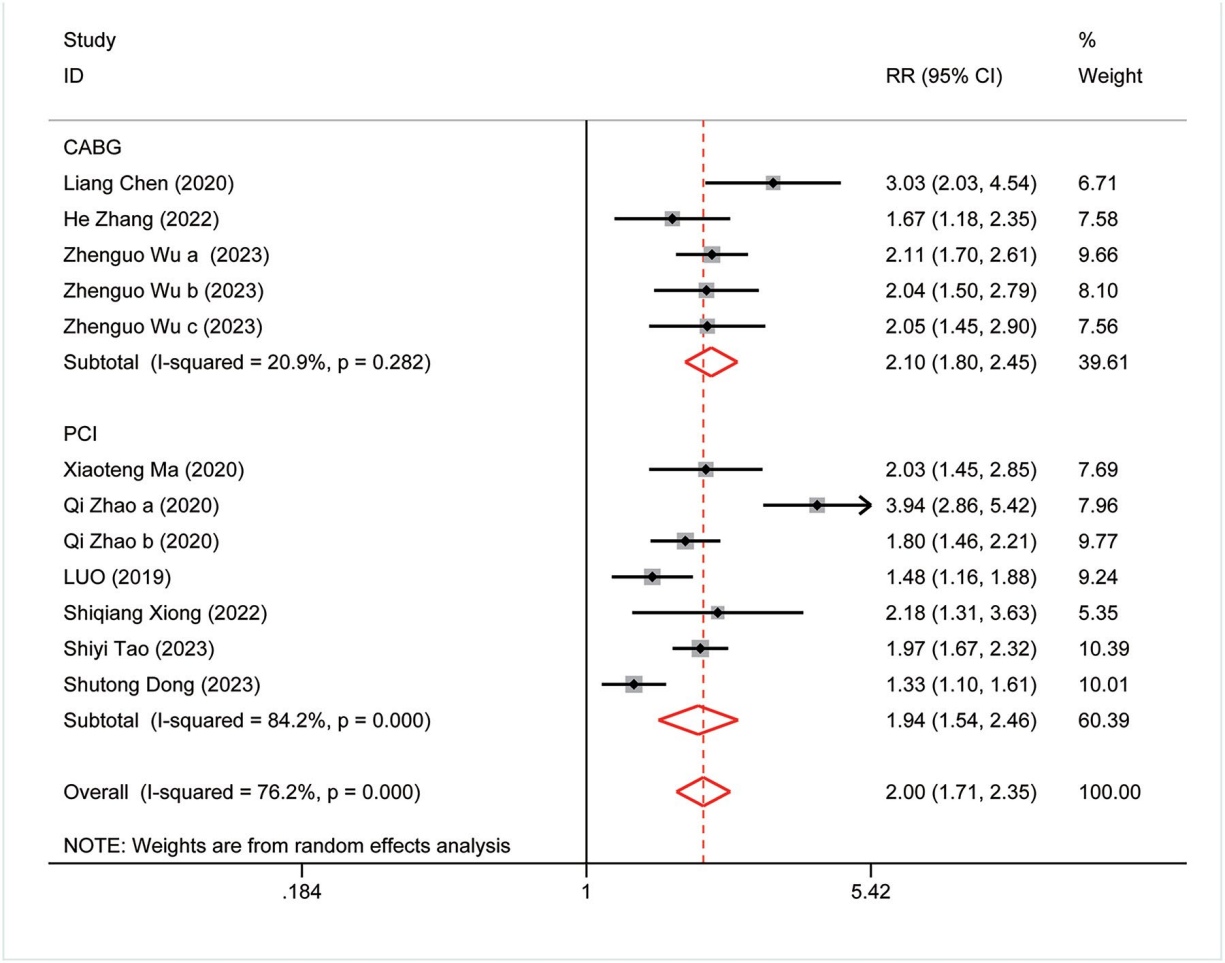
Forest plots for the meta-analysis of the association between TyG index and risk of subsequent MACEs in Patients with coronary revascularization.

The association between the TyG index and the occurrence of MACEs was explored across 12 cohort studies. Of these, 5 studies examined the relationship between the TyG index and post-CABG, while 7 studies investigated its association with post-PCI. Findings indicated an independent association between elevated TyG index and an increased risk of MACEs (RR:2.0, 95%CI:1.71–2.35, *I^2^*=76.2%, *p* < 0.0001). Subgroup analysis, based on predefined criteria, investigated the predictive role of the TyG index for MACEs after various coronary revascularization procedures. The results revealed that patients in the high TyG group had a higher probability of experiencing MACEs after both types of coronary revascularization compared to those in the low TyG group: CABG group (RR: 2.10, 95%CI:1.80–2.45, *I^2^*=20.9%, *p* = 0.0001). PCI group: (RR:1.94, 95%CI:1.54–2.46, *I^2^*=84.2%, *p <* 0.00001) (Figure 2). The results of subgroup analysis based on whether the included population had diabetes and ACS were shown in Figure SI and Figure SII.

### 3.3. Secondary outcomes

**Figure 3.**
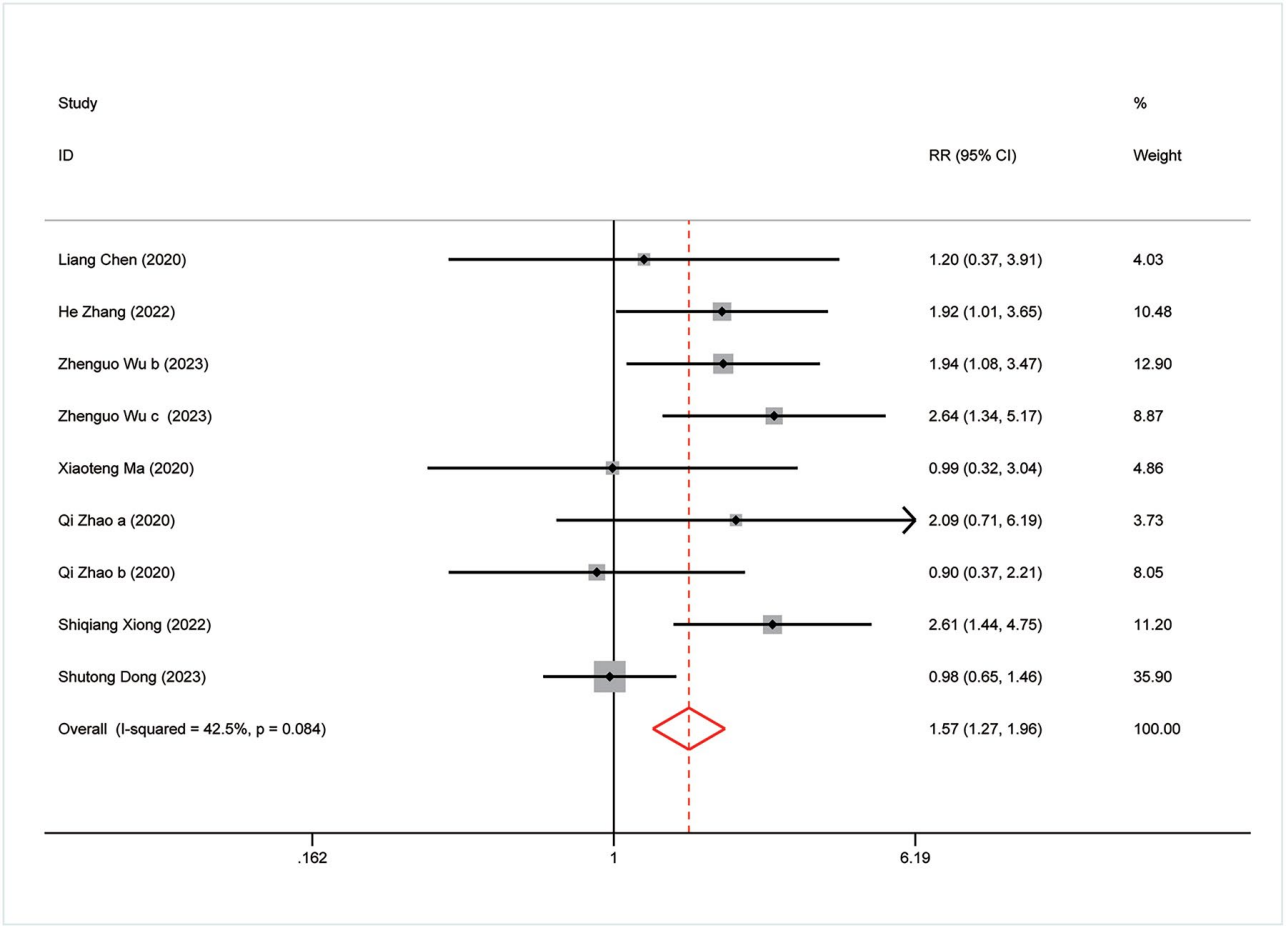
Forest plots for the meta-analysis of the association between TyG index and risk of subsequent all-cause mortality in Patients with coronary revascularization.

**Figure 4.**
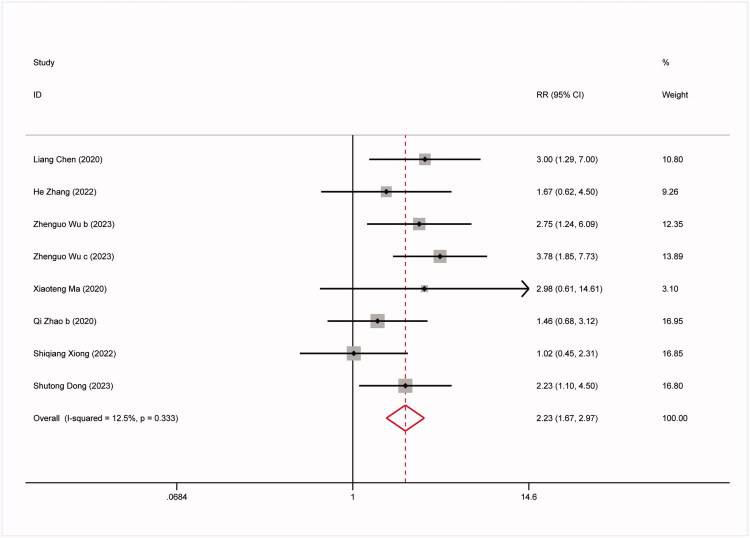
Forest plots for the meta-analysis of the association between TyG index and risk of subsequent non-fatal stroke in Patients with coronary revascularization.

**Figure 5.**
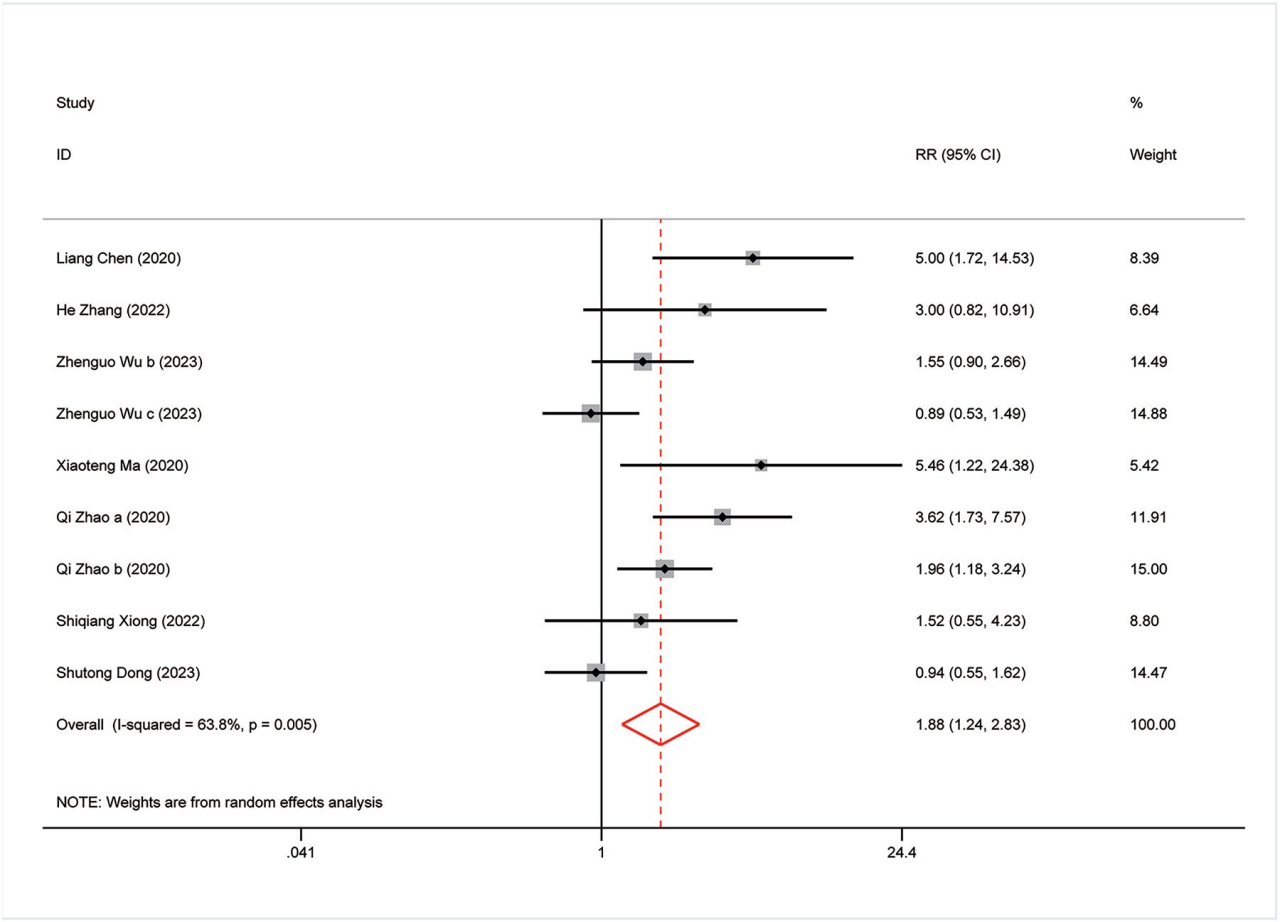
Forest plots for the meta-analysis of the association between TyG index and risk of subsequent non-fatal myocardial infarction in Patients with coronary revascularization.

**Figure 6.**
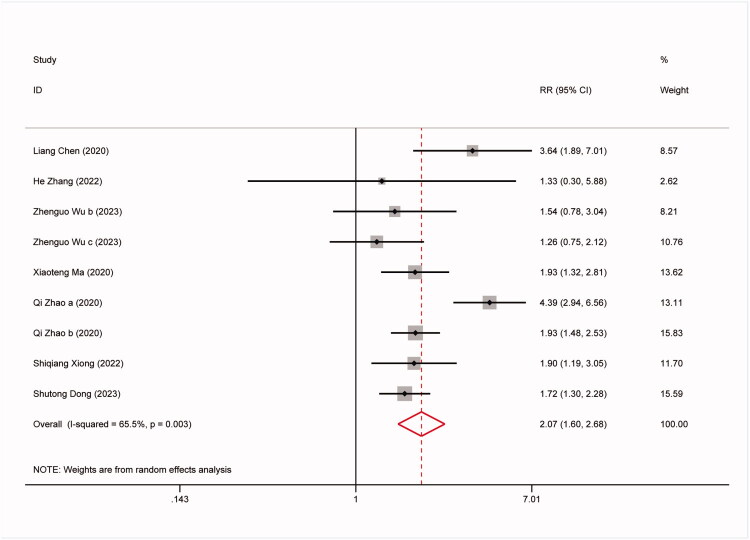
Forest plots for the meta-analysis of the association between TyG index and risk of subsequent repeat revascularization in Patients with coronary revascularization.

The study conducted further analysis on the predictive capability of the TyG index for all-cause mortality, non-fatal stroke, non-fatal myocardial infarction, and symptomatic graft failure. The results are as follows: 9 studies involved all-cause mortality: (RR:1.57,95%CI:1.27–1.96, *I^2^*=42.5%, *p* = 0.003) (Figure 3). 8 studies involved non-fatal stroke (RR: 2.23,95%CI: 1.67–2.97, *I^2^*=12.5%, *p* < 0.0001) (Figure 4). 9 studies involved non-fatal myocardial infarction: (RR:1.88,95%CI:1.24–2.83, *I^2^*=63.8%, *p* = 0.003) (Figure 5). 9 studies involved repeat revascularization: (RR:2.07,95%CI:1.60–2.68, *I^2^*=65.5%, *p* < 0.0001) (Figure 6). The findings suggest that individuals in the high TyG group are at a heightened risk of cardiovascular adverse events compared to those in the low TyG group. Subgroup analysis provided additional confirmation of the robustness of our findings (Table 3).

**Table 3.** Subgroup analysis of secondary outcomes by different coronary revascularization methods.

	Number of studies	RR	95% CI	Heterogeneity between studies (%)
All-cause mortality				
CABG	4	2.02	1.43–2.86	I^2^=0
PCI	5	1.32	0.99–1.75	I^2^=54.6
Non-fatal stroke				
CABG	4	2.90	1.93–4.36	I^2^=0
PCI	4	1.65	1.09–2.50	I^2^=0
Non-fatal myocardial infarction				
CABG	4	1.84	0.91–3.70	I^2^=70.2
PCI	5	1.97	1.12–3.48	I^2^=64.5
Repeat revascularization				
CABG	4	1.81	1.05–3.13	I^2^=54.8
PCI	5	2.19	1.61–2.68	I^2^=74.7

PCI: Percutaneous coronary intervention; MACEs: Major adverse cardiovascular events; CABG: Coronary artery bypass graft; RR:relative risk; CI: confidence interval.

### 3.4. Sensitivity analysis and publication bias

**Figure 7.**
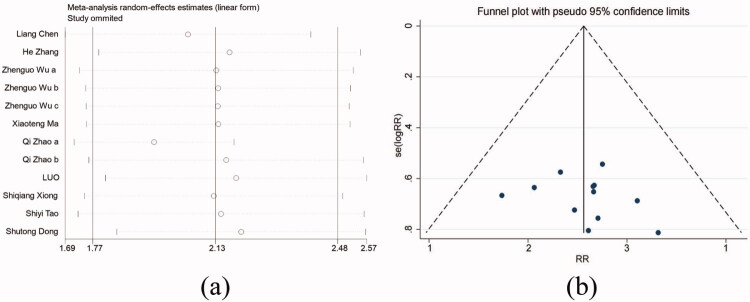
(a) Sensitivity analysis of was performed for 12 studies; (b) Funnel plot results of 12 studies according to the risk of MACEs.

Sensitivity analysis for primary outcome measures revealed that excluding any study did not substantially reduce heterogeneity or significantly impact the summarized risk ratio (RR) (Figure 7a). A bias assessment was performed for the literature included in the primary outcome measures, and the funnel plot exhibited symmetry (Figure 7b). Egger’s test, with a p-value of 0.159 (>0.05), suggested no significant publication bias As the literature included in the secondary outcome measures comprised fewer than ten studies, a publication bias test was not performed.

## 4. Discussion

The occurrence of MACEs is the main factor limiting the benefit of patients after coronary revascularization. Hence, it is imperative to identify risk markers that predict MACEs following coronary revascularization surgery and formulate corresponding preventive strategies. Analyzing this pertinent cohort study revealed a significantly elevated risk of MACEs in the high TyG group compared to the low TyG group (RR:2.0, 95%CI: 1.71–2.35, *I^2^*=76.2%, *p* < 0.00001). Moreover, Results were consistent whether CABG or PCI was performed and whether diabetes and ACS were combined. Additional analysis encompassing all-cause mortality, non-fatal stroke, non-fatal myocardial infarction, and repeat revascularization that individuals in the high TyG group faced an increased risk of cardiovascular adverse events. Hence, this study asserts that the TyG index can serve as an effective predictive factor for the occurrence of subsequent MACEs following coronary revascularization surgery.

Currently, multiple studies suggest a close association between the TyG index and the progression of coronary disease. Kahui Park et al. discovered a significant correlation between the TyG index and the progression of coronary calcification [33]. Likewise, in a 2020 study, Javad et al. identified a correlation between an elevated TyG index and the severity of coronary artery disease in patients with coronary heart disease [34]. Park GM et al.’s study suggested that as the TyG index increases, the incidence of coronary heart disease also rises [35]. These studies may, to some extent, elucidate the connection between the TyG index and adverse cardiovascular events following coronary revascularization. From a pathophysiological standpoint, the TyG index is a composite measure comprising triglycerides and fasting blood glucose. Triglycerides and fasting blood glucose are linked to the insulin resistance levels of adipocytes and the liver, respectively [36]. Currently, a wealth of evidence suggests that insulin resistance is closely intertwined with inflammation, endothelial dysfunction, coagulation imbalance, oxidative stress, poor myocardial reperfusion, microcirculatory dysfunction, plaque vulnerability, and cardiovascular remodelling [37–41]. The TyG index can, to some extent, provide a comprehensive reflection of the degree of insulin resistance in the entire body. A study by Hu et al. suggests that the TyG index can effectively predict cardiovascular risk in patients with acute coronary syndrome after PCI [42]. Subsequent meta-analysis demonstrates a positive correlation between the elevation of the TyG index and the risk of acute coronary syndrome events [43]. This aligns with our study findings and further substantiates the predictive role of the TyG index in adverse cardiovascular events following coronary revascularization.

Coronary artery revascularization is mainly performed through two methods: CABG and PCI. To gain a deeper understanding of the predictive effect of the TyG index for MACEs after these two surgeries, we conducted a predefined subgroup analysis. The study results indicate that the TyG index exhibited good predictive performance in both the CABG group (RR: 2.10, 95% CI: 1.80–2.45, *I^2^* = 20.9%, *p* = 0.0001) and the PCI group (RR: 2.0, 95% CI: 1.71–2.35, *I^2^* = 84.2%, *p* < 0.00001). However, we observed higher heterogeneity in the PCI group. Analysis revealed that CABG is typically employed for multi-vessel coronary artery disease, suitable for more complex cases, especially those with severe left main coronary artery (LM) disease or multi-vessel disease [44]. In contrast, PCI is suitable for simpler lesions, especially single-vessel coronary artery disease. With the development of drug balloons and stents, PCI has become a primary treatment strategy for acute coronary syndrome (ACS) [45], and it demonstrates comparable advantages to CABG in the treatment of many complex lesions. This study suggests that the higher heterogeneity in the PCI group may be due to the inclusion of patients with ACS, CAD, and acute myocardial infarction, leading to differences in patient characteristics, TyG patterns, and study designs. Therefore, future research aims to include larger and more diverse samples and conduct separate analyses for the two different methods of revascularization to obtain more accurate results.

Moreover, subgroup analysis reveals consistent findings irrespective of the presence of diabetes or ACS in the population. Elevated TyG index is closely linked to the development of diabetes and hypertension. Ren et al. demonstrated the pivotal role of the TyG index in linking the Chinese visceral adiposity index (CVAI) with hypertension and diabetes among elderly individuals [46]. Zhang et al.’s study demonstrated that as the triglyceride glucose index increases, the cumulative risk of type 2 diabetes mellitus in normal-weight individuals also increases accordingly [47]. Type 2 diabetes is significantly associated with the occurrence, severity, and prognosis of coronary heart disease [48,49]. Laura et al. demonstrated that diabetes and hyperlipidemia influence the applicability of the TyG index in patients with cardiovascular diseases [6]. However, Schwartz et al.’s study found that while fasting triglycerides and glucose are linked to long-term and short-term cardiovascular risk following acute coronary syndrome, they are not associated with diabetes status [50]. Additionally, some studies have demonstrated a significant association between the TyG index and the occurrence of cardiovascular diseases, irrespective of diabetes and other cardiovascular risk factors [13,51]. Likewise, the findings of Liang Chen 2020, Xiaoteng Ma 2020, He Zhang 2020, and Qi Zhao et al. 2020, as included in this article, indicate that following adjustment for confounders, the predictive capacity of the TyG index for cardiovascular adverse events in patients undergoing coronary revascularization remains unaffected by diabetes status. Consequently, we anticipate further research investigating the specific influence and effectiveness of diabetes and hyperlipidemia on the relationship between the TyG index and cardiovascular risk in the future.

We attempted to further study the role of the TyG index in predicting all-cause death, non-fatal stroke, non-fatal myocardial infarction and repeat revascularization after coronary revascularization. Our findings indicate that individuals in the high TyG group face a heightened likelihood of experiencing these adverse events compared to those in the low TyG group. In a prospective cohort study, participants with a higher TyG index exhibited a twofold higher risk of myocardial infarction compared to the low TyG index group [52]^.^ In a retrospective observational study involving the general population, participants in the highest TyG index group had a 51% higher risk of all-cause mortality compared to those in the lowest TyG index group [53]. Zhang et al. discovered that a high TyG index level was linked to a higher incidence of acute myocardial infarction, a larger infarct size, and a higher rate of coronary revascularization [54]. Early meta-analysis also suggested that, among participants without baseline atherosclerotic cardiovascular disease, a higher TyG index was associated with a 26% increased risk of stroke [55]. According to the study by Laxy M et al. ST-elevation myocardial infarction (STEMI) individuals with a high TyG index were more likely to experience cardiogenic death, malignant arrhythmias, unstable angina, and heart failure re-hospitalization within 2 years after PCI [56], aligning closely with our study results.Research indicates that elevated triglycerides (TG) and fasting plasma glucose (FPG) contribute to the damage of coronary artery endothelial cells, activation of platelets, release of inflammatory mediators, promotion of atherosclerosis, and collectively drive the occurrence and development of cardiovascular diseases. Fluctuations in glucose increase oxidative stress, inflammatory cytokines, endothelial dysfunction, and excessive activation of the sympathetic nervous system [57–59], contributing to the occurrence of adverse cardiovascular events. As mentioned earlier, the TyG index serves as a useful marker for identifying insulin resistance and metabolic disorders [60,61]. Combining our study results further corroborates the significant value of the TyG index in the prognostic assessment of all-cause mortality, non-fatal stroke, non-fatal myocardial infarction, and symptomatic graft failure.

Presently, a growing number of patients undergo coronary revascularization surgery; nevertheless, a significant proportion still experiences a high incidence of adverse cardiovascular events postoperatively. Hence, there is a pressing need to investigate new predictive factors for the early identification of high-risk patients and implement interventions. This approach aims to decrease the incidence of adverse cardiovascular (CV) events postoperatively and enhance prognosis. There are various risk prediction models for revascularization in clinical practice, with commonly used ones including the Society of Thoracic Surgeons (STS) score and the SYNTAX score [62]. However, the STS score encompasses 40 clinical indicators and 2 coronary angiography indicators, primarily employed to predict the 30-day mortality after CABG. The SYNTAX score requires calculating risk scores based on the anatomical characteristics of coronary artery lesions, mainly used for long-term (≥1 year) prediction of major adverse cardiovascular and cerebrovascular events in patients with left main or triple-vessel disease after PCI. In comparison to the aforementioned two methods, acquiring the TyG index is more convenient, straightforward, and cost-effective. It can be directly obtained in clinical practice, thereby aiding clinicians in promptly and efficiently identifying high-risk patients post-coronary revascularization surgery. In the future, the TyG index can be integrated into the risk stratification system for patients undergoing coronary revascularization. Measuring the TyG index of patients can guide risk management for high-risk individuals, ameliorate postoperative symptoms, and enhance overall quality of life.

This study also has some limitations. Firstly, the studies available for meta-analysis are restricted. Further investigation is needed to explore the impact of other study characteristics on prognosis, including the population undergoing coronary revascularization and the duration of follow-up. Secondly, the existence of a linear relationship between the TyG index and the risk of MACE is unclear. Given the current results of the meta-analysis and previous studies, determining the optimal cutoff value of the TyG index as a prognostic factor for patients undergoing coronary revascularization is challenging. Future research is needed to address this issue. Thirdly, all studies in the article are related to China, so our findings need to be validated in populations from different regions. Guidelines for the TyG index in different populations should also be formulated based on local evidence. We look forward to the inclusion of more studies with larger sample sizes from different regions in the future to increase the representativeness of the results. Additionally, we cannot ascertain the differences in diet and nutritional factors, and whether the use of glucose-lowering and lipid-lowering drugs before hospitalization will impact the collection of the TyG index, potentially influencing the relationship between the TyG index and the subsequent incidence of MACEs.Lastly, all included studies measured the TyG index only once. The stability of this index, the changes in the TyG index during hospitalization and after discharge in ACS patients, and its relationship with the subsequent incidence of MACEs require further investigation.

## 5. Conclusions

In conclusion, current evidence from cohort studies suggests that higher TyG index may be an independent predictor of subsequent risk of MACEs in patients with coronary revascularization. Additionally, the TyG index is cost-effective and clinically user-friendly. It can be integrated into the risk stratification system for patients post-coronary revascularization in clinical practice, thereby guiding individualized treatment for patients. This will be the focus of future research.

## Supplementary Material

Supplemental Material

Supplemental Material

Supplemental Material

PRISMA_2020_checklist.docx

## Data Availability

The data supporting the findings of this study are available from the corresponding author upon reasonable request.
