# Supplementary material for: Triglyceride-glucose index as a novel predictor of major adverse cardiovascular events in patients with coronary revascularization: a meta-analysis of cohort studies
Source: Ann Med. 2025 Dec 27;58(1):2607796. doi: 10.1080/07853890.2025.2607796 (PMC12777766; doi:10.1080/07853890.2025.2607796)
Supplement: Supplemental Material [file IANN_A_2607796_SM4575.docx]

Figure SI：(a) Forest plot for MACE in patients with diabetes. (b) Forest plot for MACE in patients without diabetes.

Figure SII：(a) Forest plot for MACE in patients with ACS. (b) Forest plot for MACE in patients without ACS.
