# Supplementary figures and images for: Triglyceride-glucose index as a novel predictor of major adverse cardiovascular events in patients with coronary revascularization: a meta-analysis of cohort studies
Source: Ann Med. 2025 Dec 27;58(1):2607796. doi: 10.1080/07853890.2025.2607796 (PMC12777766; doi:10.1080/07853890.2025.2607796)

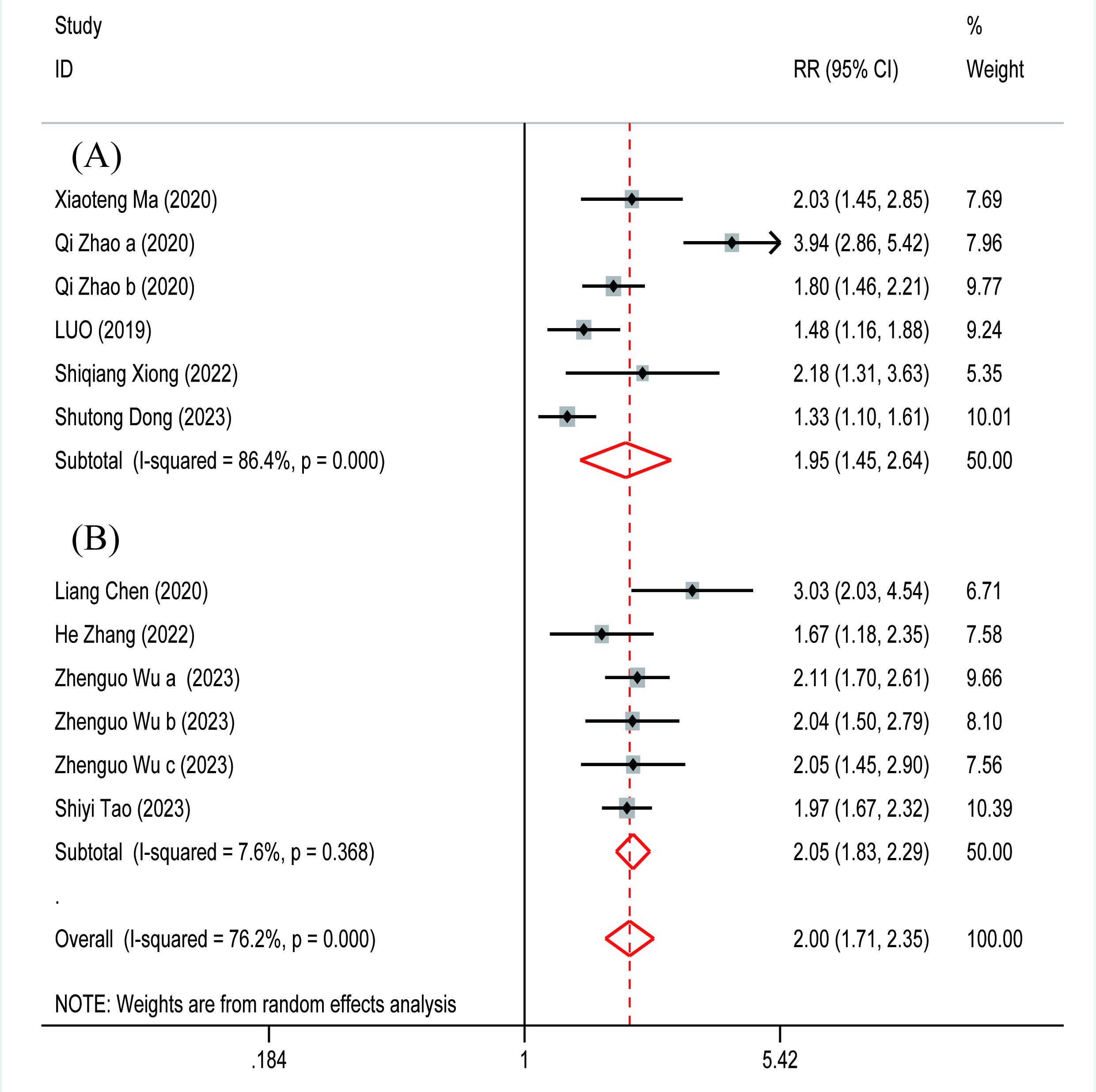

Supplement: Supplemental Material [file IANN_A_2607796_SM4557.tif]

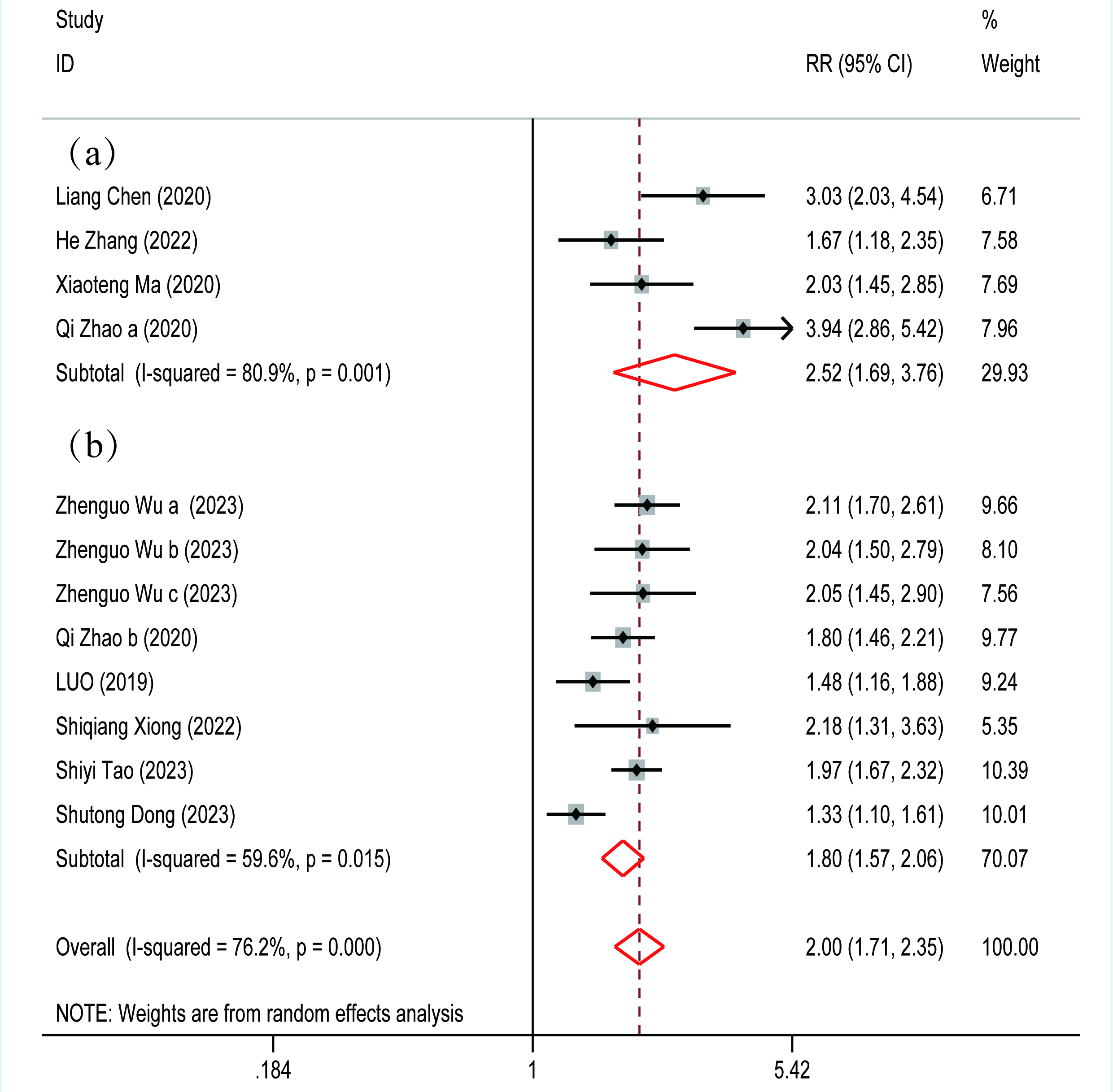

Supplement: Supplemental Material [file IANN_A_2607796_SM4554.tif]
